# Motility increase of adherent invasive *Escherichia coli* (AIEC) induced by a sub-inhibitory concentration of recombinant endolysin LysPA90

**DOI:** 10.3389/fmicb.2022.1093670

**Published:** 2022-12-22

**Authors:** Yoon Jung Hwang, Jaehak Jo, Eunsuk Kim, Hyunjin Yoon, Hyewon Hong, Min Soo Kim, Heejoon Myung

**Affiliations:** ^1^Department of Bioscience and Biotechnology, Hankuk University of Foreign Studies, Yong-In, Gyung-Gi Do, South Korea; ^2^LyseNTech Co. Ltd., Seongnam, Gyung-Gi Do, South Korea; ^3^Department of Molecular Science and Technology, Ajou University, Suwon, Gyung-Gi Do, South Korea

**Keywords:** bacteriophage, endolysin, flagella, AIEC, membrane stress

## Abstract

Endolysins are bacteriophage enzymes required for the eruption of phages from inside host bacteria *via* the degradation of the peptidoglycan cell wall. Recombinant endolysins are increasingly being seen as potential antibacterial candidates, with a number currently undergoing clinical trials. Bacteriophage PBPA90 infecting *Pseudomonas aeruginosa* harbors a gene encoding an endolysin, *lys*PA90. Herein, recombinant LysPA90 demonstrated an intrinsic antibacterial activity against *Escherichia coli in vitro*. It was observed that a sub-inhibitory concentration of the recombinant protein induced the upregulation of genes related to flagella biosynthesis in a commensal *E. coli* strain. Increases in the number of bacterial flagella, and in motility, were experimentally substantiated. The treatment caused membrane stress, leading to the upregulation of genes *rpo*E, *rpo*H, *dna*K, *dna*J, and *flh*C, which are upstream regulators of flagella biosynthesis. When adherent invasive *Escherichia coli* (AIEC) strains were treated with subinhibitory concentrations of the endolysin, bacterial adhesion and invasion into intestinal epithelial Caco-2 cells was seen to visibly increase under microscopic examination. Bacterial counting further corroborated this adhesion and invasion of AIEC strains into Caco-2 cells, with a resultant slight decrease in the viability of Caco-2 cells then being observed. Additionally, genes related to flagella expression were also upregulated in the AIEC strains. Finally, the enhanced expression of the proinflammatory cytokine genes TNF-α, IL-6, IL-8, and MCP1 in Caco-2 cells was noted after the increased invasion of the AIEC strains. While novel treatments involving endolysins offer great promise, these results highlight the need for the further exploration of possible unanticipated and unintended effects.

## 1. Introduction

Bacteriophages use the phage-encoded enzymes holin and endolysin to burst out from inside host bacteria (Young, 2014). Holin makes holes in the plasma membrane, and endolysin degrades the peptidoglycan cell wall (Donovan, 2007). When recombinant endolysins are externally provided, they can act as antibacterials (Schmelcher et al., 2012). However, the presence of an outer membrane in Gram-negative bacteria usually hinders the antibacterial activity of recombinant endolysins. There have been cases where the antibacterial efficacies of native or engineered endolysins were observed and reported. For example, based on the endolysin of *Pseudomonas fluorescens* phage OPBA, LoGT-001 is a polycationic nonapeptide-fused endolysin which demonstrated a 2.6 log reduction of viable bacteria within 30 min (Briers et al., 2014). Also, a C-terminal fusion of hydrophobic amino acids to Lysep3, an *Escherichia coli* phage endolysin, led to an increase in bactericidal activity (Yan et al., 2019). In addition, a Salmonella phage SPN9CC-encoded endolysin exhibited broad-spectrum antibacterial activity against *S. typhimurium*, *E. coli*, and *P. aeruginosa* (Lim et al., 2014). Furthermore, endolysin EC340 derived from an *E. coli* phage possessed broad-spectrum antibacterial activity against Gram-negative bacteria, an activity which increased with the N-terminal fusion of an antimicrobial peptide, cecropin A (Hong et al., 2022).

Bacteria are known to use a variety of strategies for movement (Miyata et al., 2020; Wadhwa and Berg, 2022). Flagella are the representative machinery for motility and they facilitate bacterial movement in stressful or harmful environments (Stock and Surette, 1996). In *E. coli*, the regulon of flagella biosynthesis consists of more than 40 genes of 3 classes (Liu and Matsumura, 1994). The *flh*D and *flh*C genes in class I serve as the master regulators (Wang et al., 2006). These two polypeptides comprise FlhD_4_C_2_ complex and serve to activate the transcription of class II flagella genes, which are related to basal body assembly and export machinery (Barker and Prüss, 2005; Wang et al., 2006). Amongst the class II genes, *fli*A encodes an RNA polymerase sigma factor which is responsible for expression of class III genes. Class III genes include those responsible for flagellin synthesis. In *E. coli*, the heat shock genes *dna*J and *dna*K are essential for flagella biosynthesis, with deletion mutants lacking *dna*J or *dna*K exhibiting 10-to 20-fold decreases in flagella synthesis (Shi et al., 1992). Expression of *flh*DC operon also decreased in the same cells (Shi et al., 1992). *rpo*H, the key regulator of heat shock response, is known to positively regulate the expression of *dna*J and *dna*K (Liberek et al., 1992).

Bacteria also respond and adapt to signaling caused by various envelope stresses. Extracytoplasmic stresses such as changes in osmolarity, pH, detergent, or viral infection, lead to envelop stresses. *rpo*E controls the regulon governing extracytoplasmic stress signaling system (Raivio, 2005; Hayden and Ades, 2008). The extracytoplasmic function (ECF) sigma factor σ*^E^* responds to outer membrane disorders in a complex signal cascade involving the degradation of the inner membrane anti-sigma factor, RseA, with the subsequent release of σ*^E^* into the cytoplasm occurring (Klein and Raina, 2019). When *rpo*E is overexpressed in *E. coli*, sigma factor *rpo*H is one of the genes whose upregulation has been observed (Bury-Moné et al., 2009).

Increased swarming motility due to changes in environmental factors or nutrition has been observed in *Proteus mirabilis* (Tuson et al., 2013), *Pseudomonas aeruginosa* (Rashid and Kornberg, 2000; Zhang et al., 2007), and *Salmonella enterica* (Toguchi et al., 2000; Das et al., 2018). Flagella-induced motility is related to bacterial colonization to host cells by cellular adhesion and invasion. When bacteria translocate from one tissue to other tissues of the host, flagella motility is essential in some cases. When *fli*C, the gene encoding flagellin from a motile enteropathogenic *E. coli* H6 strain, was expressed in a nonmotile *E. coli* K12 strain, bacterial adhesion of the K12 strain to HeLa cells was observed (Girón et al., 2002). When *fli*C was deleted from an enterotoxigenic *E. coli*, bacterial adherence to Caco-2 cells decreased (Roy et al., 2009). Osmotic pressure induced an increase in flagella expression, in turn leading to an increase in motility in *Escherichia albertii* (Ikeda et al., 2020). The flagellated bacteria then invaded Caco-2 cells in the presence of gentamycin, resulting in increased bacterial survival. *E. coli* has been known to move in liquid media or semisolid media using flagella in response to external signals, which confers advantages for growth and survival (Baker et al., 2006).

There have been reports that exposure of bacteria to subinhibitory concentrations of antibiotics diminishes flagella synthesis and/or function. Azithromycin, a macrolide antibiotic, was able to affect the formation of flagellar filaments, specifically by reducing the amount of flagellin exported from *S. enterica*, though it was not involved in the suppression of the synthesis of flagellin (Matsui et al., 2005). Similarly, disruption of flagellar assembly or function in *P. aeruginosa* was observed when the bacteria were exposed to vancomycin, perhaps by way of conferring a fitness advantage to the bacteria (Rundell et al., 2020). Also, the swarming motility of *S. enterica* was impaired in the presence of sub-inhibitory concentrations of antibiotics cefotaxime, ciprofloxacin, trimethoprim, or chloramphenicol, but not that of amikacin, colistin, kanamycin or tetracycline (Irazoki et al., 2017). In the latter study, it was particularly noted that chloramphenicol-treated *S. enterica* cells exhibited clear decreases in their flagellar content.

With a number of endolysins being in either preclinical or clinical development as antibacterials, an exploration of the possible effects of recombinant endolysins on bacterial physiology is warranted. Once recombinant endolysins are introduced *in vivo*, concentrations would decrease over time, becoming sublethal in parts of the body where pathogenic bacteria still reside. In this study, we therefore present a gene expression profile in the presence of sublethal doses of a recombinant endolysin and explore its biological significance.

## 2. Materials and methods

### 2.1. Bacterial strain bacteriophage

*Escherichia coli* ATCC8739 was purchased from the American Type Culture Collection. Adherent invasive *E. coli* (AIEC) strains were kindly gifted by Professor Christel Neut (Rahmouni et al., 2018). All strains were cultured in Luria-Bertani (LB) media. Bacteriophage PBPA90 infecting *P. aeruginosa* was obtained from the Bacteriophage Bank of Korea. The bacteriophage was isolated from Opo Wastewater Facility in Gwangju, Gyeonggi-do, South Korea. The host strain used for the isolation was *P. aeruginosa* (ATCC13388). An endolysin gene was annotated and named LysPA90 (GenBank accession no. MW815133).

### 2.2. Construction of deletion mutant bacteria

Deletion strains were constructed using an FRT/FLP recombinase system previously discussed (Hoang et al., 1998). Plasmids pKD4, harboring the FRT-kanamycin resistance gene-FRT region (FRT cassette), pKD46 for homologous recombination, and pCP20 expressing flippase, were used. The target genes deleted from *E. coli* ATCC8739 were; *rpo*E (GenBank WP_001295364.1 (2,380,427 ~ 2,381,002 bp)), *rpo*H (GenBank WP_000130217.1 (3,288,345 ~ 3,289,199 bp)), *flh*C (GenBank WP_001291603.1 (1,561,600 ~ 1,562,178 bp)), and *flh*D (GenBank WP_001295647.1 (1,562,181 ~ 1,562,531 bp)). A sewing PCR generated left arm (500 bp) upstream of the target gene-FRT kanamycin cassette - right arm (500 bp) downstream of the target gene and the product was cloned into pET21a plasmid at the BamHI and HindIII sites. The insert was PCR amplified and 500 ng of the product was electroporated into *E. coli* ATCC8739 harboring pKD46. The transformant was screened using LB media containing kanamycin. Selected deleted bacterial colonies were transformed with pCP20 followed by induction of FLP at 45°C to remove the FRT region. Colonies losing kanamycin resistance were selected and subjected to DNA sequencing for confirmation (Supplementary Figure S1).

### 2.3. Cloning, expression, and purification of PA90 endolysin

The gene encoding endolysin LysPA90 was cloned in pET21a and the plasmid was expressed in *E. coli* BL21 (DE3) pLysS. The cell was cultured in LB broth until it reached 0.5 OD^600^, followed by the addition of isopropyl-b-D-thio-Galactopyranoside (IPTG) at a final concentration of 1 mM. After 3 h of incubation, cells were harvested by centrifugation at 10,000 ×*g* for 20 min. The supernatant was discarded and the pellet was resuspended in 100 ml lysis buffer (20 mM Tris–HCl, pH 7.5, 0.5 M NaCl, 10 mM imidazole). Then the mixture was sonicated for 10 min, followed by centrifugation at 13,000 ×*g* for 40 min. The supernatant was collected and subjected to FPLC (Akta Go Fast FPLC, Cytiva, United States) with His Trap HP column. Elution was performed with 20 mM Tris–HCl, pH7.5 0.5 M NaCl buffer, at imidazole gradients of between 10 mM and 500 mM.

### 2.4. Zymogram assay

Bacterial cells were grown to the exponential phase (OD_600_ = 0.6) in fresh LB media and harvested. The cells were washed once with a wash buffer (20 mM Tris–HCl, pH 7.5) and harvested by centrifugation at 4,000 ×*g* for 15 min and the pellets resuspended in 1 ml of Tris buffer. Cells were autoclaved and added to a 15% SDS-PAGE gel before polymerization. Then, 3 μg of purified endolysin was mixed with 2X sample buffer (0.5 mM Tris–HCl, pH 6.8, 20% glycerol, 0.2% bromophenol blue), and subjected to SDS-PAGE. After electrophoresis, the gel was washed in deionized water (DW) for 1 h and incubated in reaction buffer (1% Triton X-100, 20 mM Tris–HCl, pH 7.5) until clear zones became visible.

### 2.5. Antibacterial efficacy test *in vitro*

The antibacterial activity of the purified protein was assessed for *E. coli* ATCC8739. Bacteria were grown to an exponential phase (OD_600_ = 0.5), washed with reaction buffer (20 mM Tris–HCl, pH 7.5), and diluted in the buffer to approximately 10^6^ cells/ml. Then, 100 μl of the bacterial suspension was mixed with 100 μl of the purified endolysin in reaction buffer and the mixture incubated at 37°C for 2 h. Finally, the mixture was diluted with PBS and plated on LB agar. After overnight incubation at 37°C, colonies were counted.

### 2.6. RNA isolation and RNA sequencing

1 × 10^6^ CFU of *E. coli* ATCC8739 was collected by centrifugation and the pellet resuspended in 1 ml of 20 mM Tris–HCl, pH 7.5 followed by the addition of lysin LysPA90 at a final concentration of 2 μM. The mixture was then incubated at 37°C for 40 min. The supernatant was discarded after centrifugation at 4,000 ×*g* for 5 min and the pellets were collected. RNA isolation from the collected bacterial cells was performed using AccuPrep^®^ Bacterial RNA Extraction Kits (Bioneer, South Korea) in accordance with the manufacturer’s protocols. RNA sequencing was performed by Macrogen (South Korea).

### 2.7. RNA-sequencing (RNS-Seq) data analysis

RNA-Seq data was normalized using Fragments Per Kilobase of transcript per Million fragments mapped (FPKM) (Trapnell et al., 2010). Genes with a log_2_ [fold change] larger than 6 or smaller than-6 were regarded as Differentially Expressed Genes (DEGs). The value of log_2_ [fold change] was defined as log_2_ [FPKM_LysPA90_/FPKM_non-treated_]. Clusters of orthologous groups (COG) analysis (Tatusov et al., 1997) was conducted to determine the proportion of DEGs in each functional group. Gitools v2.2.2 was employed to display the alteration in gene expression using heat maps (López Bigas and Pérez Llamas, 2011).

### 2.8. Real time RT-PCR

For cDNA synthesis, 5 μg of isolated RNA, 1,000 units of reverse transcriptase (DyneBio, South Korea), and random hexamer were mixed and incubated at 42°C for 5 min followed by a further incubation at 50°C for 60 min. Heat inactivation of the mixture was performed by incubation at 70°C for 15 min. Afterwards, the cDNAs, proper primers for each target gene, and SYBR Green qPCR 2X mix (DyneBio, South Korea) were mixed, with the reaction performed in QuantStudio5 (Applied Biosystems, United States).

### 2.9. Western blot with anti-FliC antibody

The anti-*E. coli* FliC antibody was purchased from Abcam, United States (cat. no. ab93713).

### 2.10. 1-N-phenylnaphthylamine (NPN) uptake assay

The methodology for the NPN uptake assay has been previously described (Helander and Mattila-Sandholm, 2000). Briefly, *E. coli* ATCC8739 were grown to 0.4 OD^600^ and harvested and resuspended in 5 mM HEPES buffer, pH 7.0. The concentration was adjusted to 10^6^ cells/ml. 100 μl of cell suspension was added to each well of a 96 well black plate. Purified endolysin and 40 μM NPN were mixed at a ratio of 1:1 (v/v), and 100 μl of the resultant mixture was added to the wells containing the bacteria. Polymyxin B (Sigma, United States) was used as a positive control. After incubation at 37°C for 5 min, fluorescence was measured by microplate reader (SpectraMax iD3, Molecular Devices, United States) with excitation at 350 nm and emission at 420 nm. The concentration of LysPA90 used for the assay was 0.5 μM.

### 2.11. Cell adhesion assay and invasion assay

1 × 10^5^ Caco-2 cells were seeded into the wells of a 12 well plate and grown to 80% confluency at 37°C in a CO_2_ incubator. Bacteria were grown to 0.7 OD^600^ and the cells were harvested and washed twice with phosphate buffered saline (PBS), followed by a final wash with DMEM media. Caco-2 cells in the 12 well plate were subjected to a change of medium with fresh DMEM without any antibiotics, followed by the addition of bacteria at the multiplicity of infection (MOI) of 1:10. After 3 h of incubation at 37°C, the cells were washed with PBS 4 times. Cells were stained with Giemsa staining solution (Sigma, United States) for 3 min and observed under a light microscope. For the quantification of adhered bacterial cells, the mixture of Caco-2 cells and bacterial cells in the 12 well plate was washed with PBS 4 times followed by treatment with 0.1% Triton X-100 in PBS for 2 min. The detached bacterial cells were counted by plating on an LB agar plate. For the quantification of invaded bacterial cells, adherent bacteria were removed by treatment with gentamycin at a concentration of 100 μg/ml in the mixture, and subsequently the same bacterial cell counting method described above was employed.

### 2.12. MTT assay

1 × 10^4^ Caco-2 cells were seeded in a well in a 96 well plate and incubated at 37°C for 24 h. 5 × 10^6^ AIEC 43 or 52 cells were preincubated with LysPA90 at 0.5 μM for 20 min, washed with DMEM, and resuspended with DMEM. The mixture was added to a well containing Caco-2 cells and the plate incubated for 6 h. 3-(4,5-dimethylthiazol-2-yl)-2,5-diphenyltetrazolium bromide (MTT) assay was performed as per the manufacturer’s instructions (Cell Viability Assay Kit, Dong-In LS, South Korea).

### 2.13. Motility assay

Swarming assay was conducted on 0.3% semi-solid agar plates. 1 × 10^6^ CFU of bacteria was collected by centrifugation and the pellet resuspended in 1 ml of 20 mM Tris–HCl, pH 7.5 followed by the addition of lysin LysPA90 at a final concentration of 2 μM. The mixture was incubated at 37°C for 40 min. The supernatant was discarded after centrifugation at 4,000 ×*g* for 5 min and the pellet was resuspended with 100 μl of PBS. 3 μl of solution was spotted on the plates and the plates were incubated for 3 h at 37°C. The plates were observed under light box to see any halo zones created.

### 2.14. Transmission electron microscopy

TEM images were obtained via TEM (Talos L120C, 120 kV, Czech) at the National Instrumentation Center for Environmental Management (Seoul National University, South Korea). Bacterial samples were dropped on glow-discharged Formvar-coated copper grids and stained with 0.5% (w/v) uranyl acetate for 1 min before taking the images.

### 2.15. Statistical analysis of data

For statistical analysis, student’s t-tests were performed using Prism version 9.3.0 (GraphPad software).

## 3. Results

### 3.1. Antibacterial activity of recombinant endolysin LysPA90

**Figure 1:**
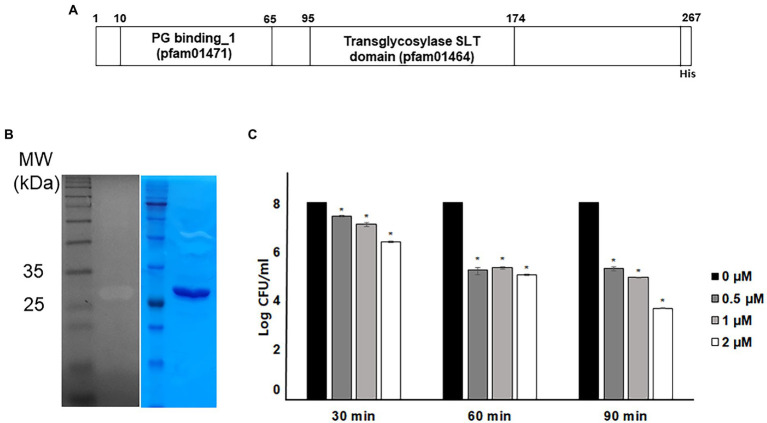
Antibacterial activity of recombinant endolysin LysPA90. **(A)** Domain structure of endolysin LysPA90. Numbers indicate amino acid positions. **(B)** Zymogram assay of purified recombinant endolysin LysPA90. Left; zymogram on *E. coli* ATCC8739, right; SDS-PAGE analysis of purified LysPA90. **(C)** Antibacterial activity of purified recombinant LysPA90 against *E. coli* ATCC8739. Antibacterial activity was tested at concentrations of 0 ~ 2 μM for 30 to 90 min. Experiments were performed in triplicate. Data are expressed as mean ± standard deviation.

The annotated endolysin gene from bacteriophage PBPA90 encoded a 260 amino-acid long protein. It harbored a peptidoglycan cell wall binding domain (pfam01471) and a transglycosylase (muramidase) domain (pfam01464; Figure 1A). The gene was expressed with a C-terminal hexahistidine tag and the overexpressed protein was purified to near homogeneity. Its lysozyme activity was confirmed in a zymogram assay (Figure 1B). The recombinant protein exhibited an antibacterial activity against *E. coli* in time-and dose-dependent manners (Figure 1C). However, the minimal inhibitory concentration (MIC) was >128 μg/ml (data not shown), suggesting the intrinsic antibacterial activity was relatively weak. As the decrease in bacterial count was minimal with the treatment of LysPA90 at 0.5 μM (14.7 μg/ml) for 30 min, these conditions were selected for further studies examining the effects of the endolysin at sub-inhibitory concentrations.

### 3.2. Upregulation of flagella-associated genes upon LysPA90 treatments

**Figure 2:**
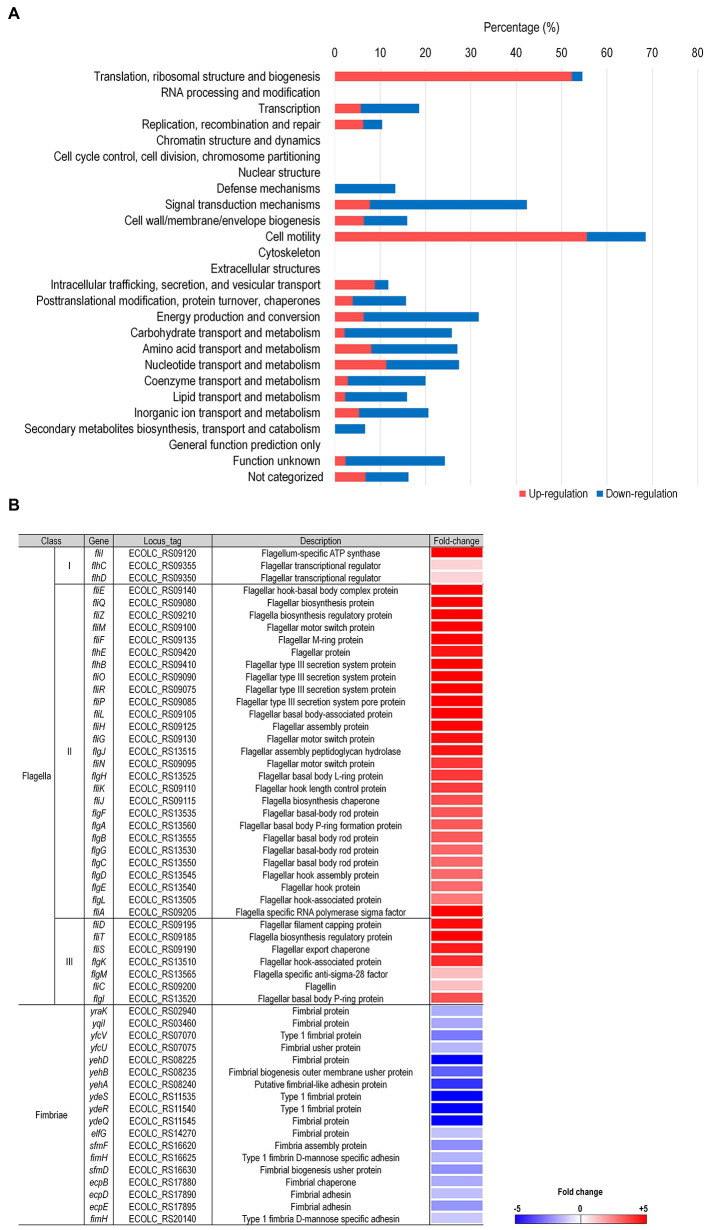
Comprehensive transcriptome analysis of *E. coli* upon treatment with LysPA90. **(A)** Differentially expressed genes (DEGs) were grouped based on COG analysis. Genes upregulated (red bars) or downregulated (blue bars) more than six-fold were functionally grouped. The number of DEGs found in each category was divided by the total number of genes in the same category and is shown as a percentile. **(B)** Genes associated with flagella and fimbriae were sorted from the RNA-Seq data and the expression ratio (LysPA90/non-treated) is displayed in a colorimetric gradient: downregulation in blue and upregulation in red.

In order to better understand bacterial physiological responses to LysPA90 treatments, transcriptomic analysis was conducted with *E. coli* strain ATCC8739 subjected to 30 min of treatment with LysPA90. Differentially Expressed Genes (DEGs) with greater than six-fold of transcriptional alterations following LysPA90 treatment were sorted, with the results listed in Supplementary Table S1. These latter DEGs, which included 203 upregulated genes and 293 downregulated genes, were categorized into 26 groups based on their predicted functions using COG analysis (Figure 2A). Genes belonging to two categories; the first associated with translation, ribosomal structure, and biogenesis (54.5%; upregulation 52.3%, downregulation 2.3%), and the second being cell motility (68.5%; 55.6% upregulation, 13.0% downregulation), had undergone significant transcriptional alterations. Interestingly, the genes from these two categories were mainly upregulated upon LysPA90 treatment, while other genes tended toward downregulation. Upregulation of genes associated with translation, ribosomal structure and biogenesis might implicate bacterial *de novo* protein synthesis against structural decomposition by LysPA90. To dissect the transcriptional response associated with cell motility, the expression of genes required for bacterial flagella and fimbriae was compared (Figure 2B). Genes involved in three-tiered transcriptional cascades, including the *flhDC* master regulator (class I), genes encoding the flagellar basal body and the alternative sigma factor, such as *fli*A (σ28, class II), and genes encoding the filament and chemotaxis machinery (class III), were all upregulated after LysPA90 treatment. Conversely, genes encoding type 1 fimbriae were downregulated upon the addition of LysPA90. Bacterial motility is a crucial virulence factor, and is required for bacterial adhesion and invasion of preferable niches, as well as for defense against hostile stimuli. Bacterial physiological alterations by LysPA90, especially concerning motile behavior, were further investigated to gain insights into the influence of LysPA90 on bacterial virulence.

### 3.3. Confirmation of transcriptional and phenotypical changes of *E. coli* treated with sub-inhibitory concentrations of the endolysin

**Figure 3:**
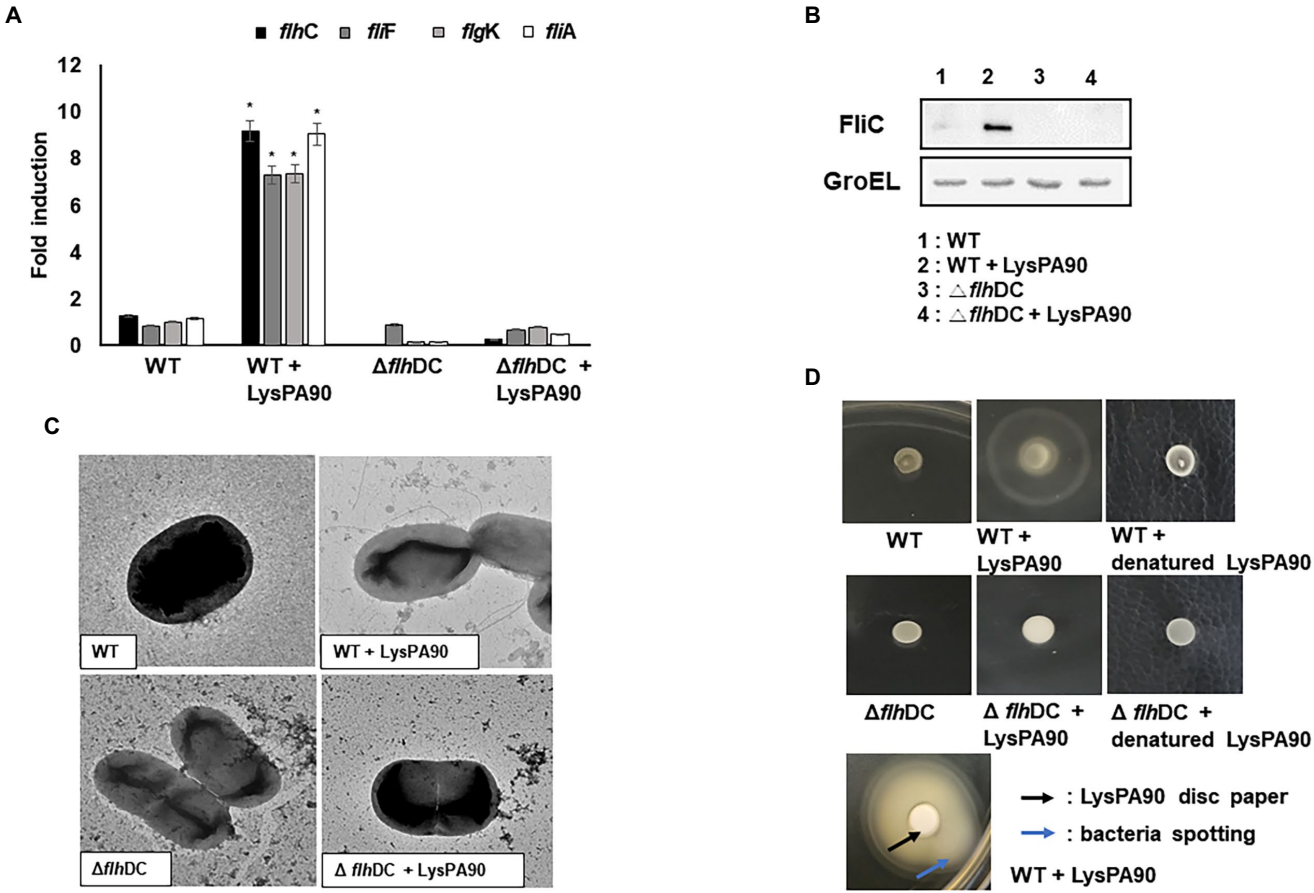
Upregulation of genes related to flagella biosynthesis and resulting phenotypical changes. **(A)** Realtime RT-PCR of genes related to flagella biosynthesis. Total RNA from wildtype (WT) or *flh*DC deletion mutant (Δ*flh*DC) strains were isolated with or without stimulation of the endolysin LysPA90. *flh*C, flagellar transcriptional regulator (Class I); *fli*A, flagella-specific sigma factor (Class II); *flg*F, flagella basal body rod protein (Class II), and *flg*K, flagella hook-associated protein (Class III). Experiments were performed in triplicate. Data are expressed as mean ± standard deviation. **(B)** Western blot analysis of FliC, flagellin protein (Class III) expression. GroEL was used as an internal control. **(C)** Transmission electron microscopic observation of flagella expression. Note that multi-flagella structure was seen only from the wildtype strain in the presence of LysPA90. **(D)** Observation of swarming motilities. Wild type (WT) or *flh*DC mutant strains (Δ *flh*DC) were spotted with or without preincubation with LysPA90 or denatured LysPA90 (top two rows). Wild type strain preincubated with LysPA90 was spotted next to a disc soaked in a solution containing 14.7 mg/ml of LysPA90 (bottom row). The latter plate was incubated longer than the former to see the halo extending beyond the disc.

The RNA-S eq results were confirmed *via* the examination of the mRNA transcription levels of selected genes through real-time RT-PCR analysis (Figure 3A). *flh*C encoding flagella transcription regulator (a Class I gene), *fli*A encoding a sigma 28 (a Class II gene), *fli*F encoding flagella M ring protein (a Class II gene), and *flg*K encoding flagella hook-associated protein (a Class III gene) were chosen for the analysis. Consistent with the RNA-Seq results, all 4 genes’ transcription increased 7- to 10-fold in the presence of sub-inhibitory concentrations of LysPA90. When the analysis was performed in a mutant *E. coli* strain lacking the *flh*C and *flh*D genes, no increase was observed with the same stimulation by the endolysin. A western blot analysis revealed that flagellin protein FliC production greatly increased in the presence of sub-inhibitory concentrations of LysPA90 (Figure 3B). Again, the increase was not seen in the mutant *E. coli* lacking the *flh*C and *flh*D genes.

An increase in the number of flagella in the presence of the sub-inhibitory concentration of LysPA90 was also observed by transmission electron microscopic analysis (Figure 3C). However, again no increase was observed with the *flhC-and flh*D deletion mutant bacteria undergoing the same stimulation.

Changes in swarming motility due to the altered expression of flagella was observed (Figure 3D). A large halo appeared after incubation of spotted wild type bacteria in the presence of LysPA90, while it was not observed in the mutant bacteria lacking *flh*C and *flh*D genes. The increase in movement was not related to bacterial escaping from the endolysin.

### 3.4. Endolysin-induced membrane stress led to upregulation of upstream regulators of flagella synthesis

**Figure 4:**
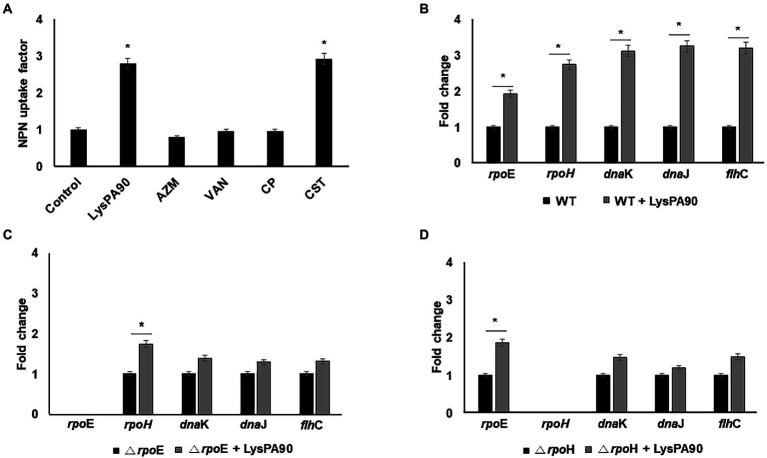
Elucidation of a possible mechanism of flagella overexpression. **(A)** Comparison of NPN uptake of bacteria in the presence of various antibacterials. Control, no antibiotics; LysPA90, endolysin (14.7 μg/ml); AZM; azithromycin (16 μg/ml), VAN; vancomycin (128 μg/ml), CP; chloramphenicol (4 μg/ml), CST; colistin (0.25 μg/ml). **(B)** Realtime RT-PCR analysis of genes involved in membrane stress in either the presence of, or in the absence of LysPA90. **(C)** Realtime RT-PCR analysis of genes involved in membrane stress from either wildtype or *rpo*E deletion mutant (Δ*rpo*E). **(D)** Realtime RT-PCR analysis of genes involved in membrane stress from either wildtype or *rpo*H deletion mutant (Δ*rpo*H). Experiments were performed in triplicate. Data are expressed as mean ± standard deviation.

Polymyxins, antimicrobial peptides effective against various Gram-negative bacteria, are known to exert their antibacterial activity through the induction of membrane stress (Ayoub Moubareck, 2020). The mechanism of action of an endolysin also seems to involve membrane stress since it enters the outer membrane and destroys the bacterial cell wall. Thus, we next examined whether LysPA90 induced membrane stress through NPN permeabilization assay (Figure 4A). LysPA90, colistin (polymyxin E), azithromycin, vancomycin, or chloramphenicol were given and outer membrane permeabilization was measured. The latter three antibiotics have been previously reported to decrease flagella expression (Matsui et al., 2005; Irazoki et al., 2017; Rundell et al., 2020) and were used as negative controls. 3-fold increases in outer membrane permeability were observed when LysPA90 or colistin was added to the bacteria, while no increase was observed with the other antibiotics. Expression of *rpo*E, known to be a response to membrane stress, increased two-fold and subsequent increases in the expression of downstream regulators including *rpo*H, *dna*K, *dna*J, and *flh*C were observed (Figure 4B). In an *rpo*E deletion mutant strain, the increase in *rpo*H expression notably diminished and no significant change in the downstream regulators of flagella synthesis was observed (Figure 4C). Additionally, in an *rpo*H deletion mutant strain, no significant change in the downstream regulators of flagella synthesis was observed (Figure 4D). We need to mention that *rpo*E or *rpo*H deletion mutant grew significantly slower than the wild type *E. coli* (Supplementary Figure S2). It was consistent with a report by Egler et al. (2005) stating that an unknown suppression had to occur for *rpo*E mutant strain to grow. Also, Calendar et al. (1988) states that *rpo*H mutant grew slowly.

### 3.5. Motility changes in adherent invasive *E. coli* (AIEC) strains in the presence of sub-inhibitory concentrations of LysPA90

**Figure 5:**
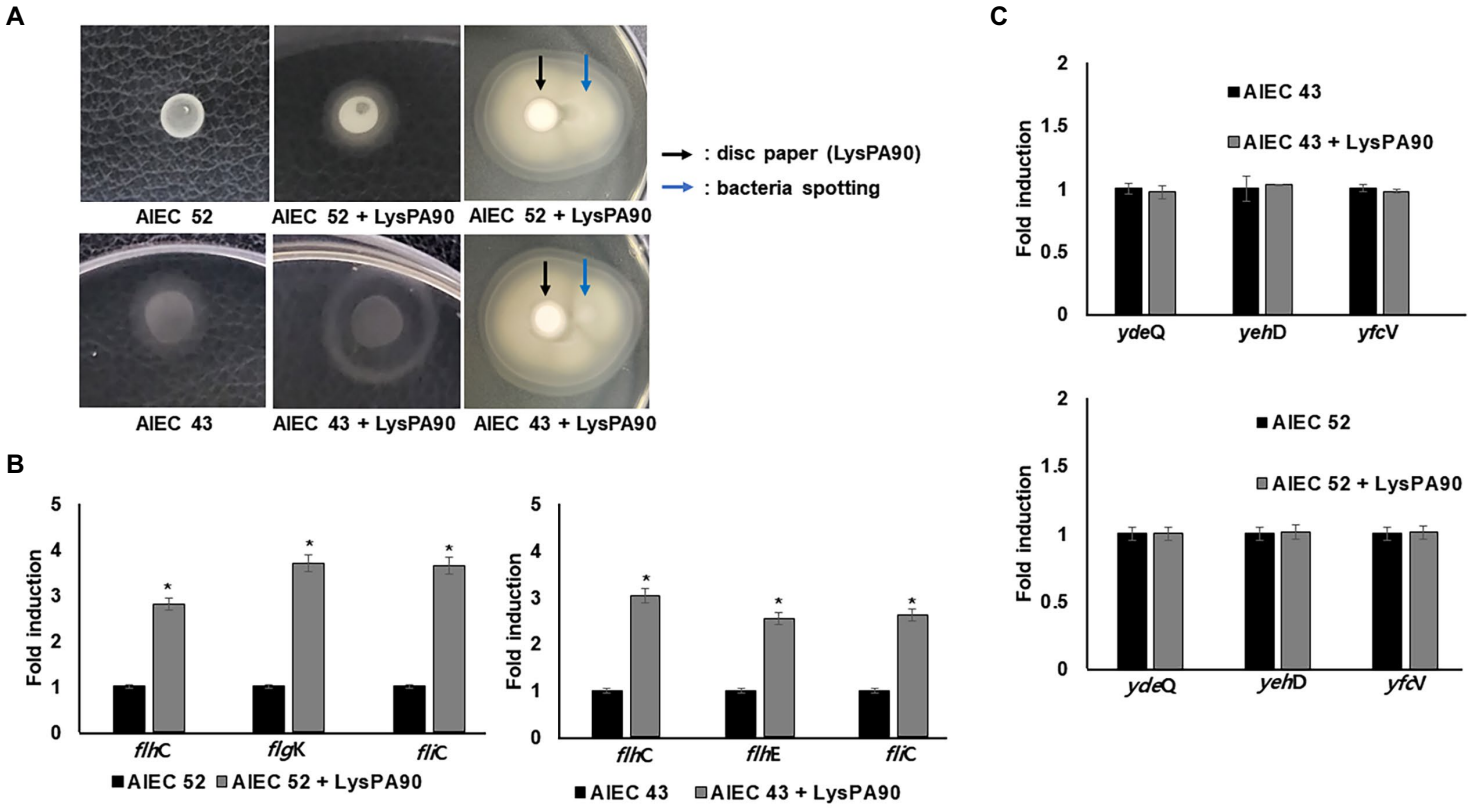
Motility increase in AIEC strains **(A)** Swarming motility of two AIEC strains, AIEC43 and AIEC52, in the presence or absence of LysPA90 (left two panels). AIEC strains preincubated with LysPA90 were spotted next to a disc soaked in a solution containing 14.7 mg/ml of LysPA90 (right panels). The latter plate was incubated longer than the former to see the halo extending beyond the disc. **(B)** Realtime RT-PCR analysis of selected genes involved in flagella biosynthesis from AIEC strains in the presence of LysPA90. *flh*C; flagellar transcriptional regulator (Class I); *flh*E, flagella protein (Class II); *fli*C, flagellin (Class III); *flg*K, flagella hook-associated protein (Class III). **(C)** Realtime RT-PCR analysis of type I fimbriae gene expression from two AIEC strains in the presence of LysPA90. *yde*Q, putative fimbrial adhesin protein; *yeh*D, putative Yeh fimbriae subunit; *yfc*V, major subunit of putative chaperon-usher fimbria. Experiments were performed in triplicate. Data are expressed as mean ± standard deviation.

Since increased motility would also increase bacterial adhesion and invasion to mammalian cells, we next checked whether motility increases were observed from AIEC strains in the presence of sub-inhibitory concentrations of LysPA90. In a swarming assay, visibly larger halos were seen from the spotted bacteria in the presence of LysPA90 (Figure 5A). However, direction of the movement was not affected by the presence of endolysin, meaning the bacteria did not escape from the endolysin. Also, an increase in expression of genes related to flagella biosynthesis (*flh*C, *fli*C, and *flh*K) was observed in the presence of LysPA90 (Figure 5B). Since type I fimbriae genes were shown to be downregulated in the RNA-Seq analysis of commensal strain *E. coli* ATCC8739 (Supplementary Table S1), we next checked whether the same transcriptional change was seen in AIEC strains (Figure 5C). The two AIEC strains underwent no changes in the transcriptional levels of type I fimbriae genes, *yde*Q, *yeh*D, and *yfc*V.

### 3.6. Increased adhesion and invasion of AIEC strains to Caco-2 cells

**Figure 6:**
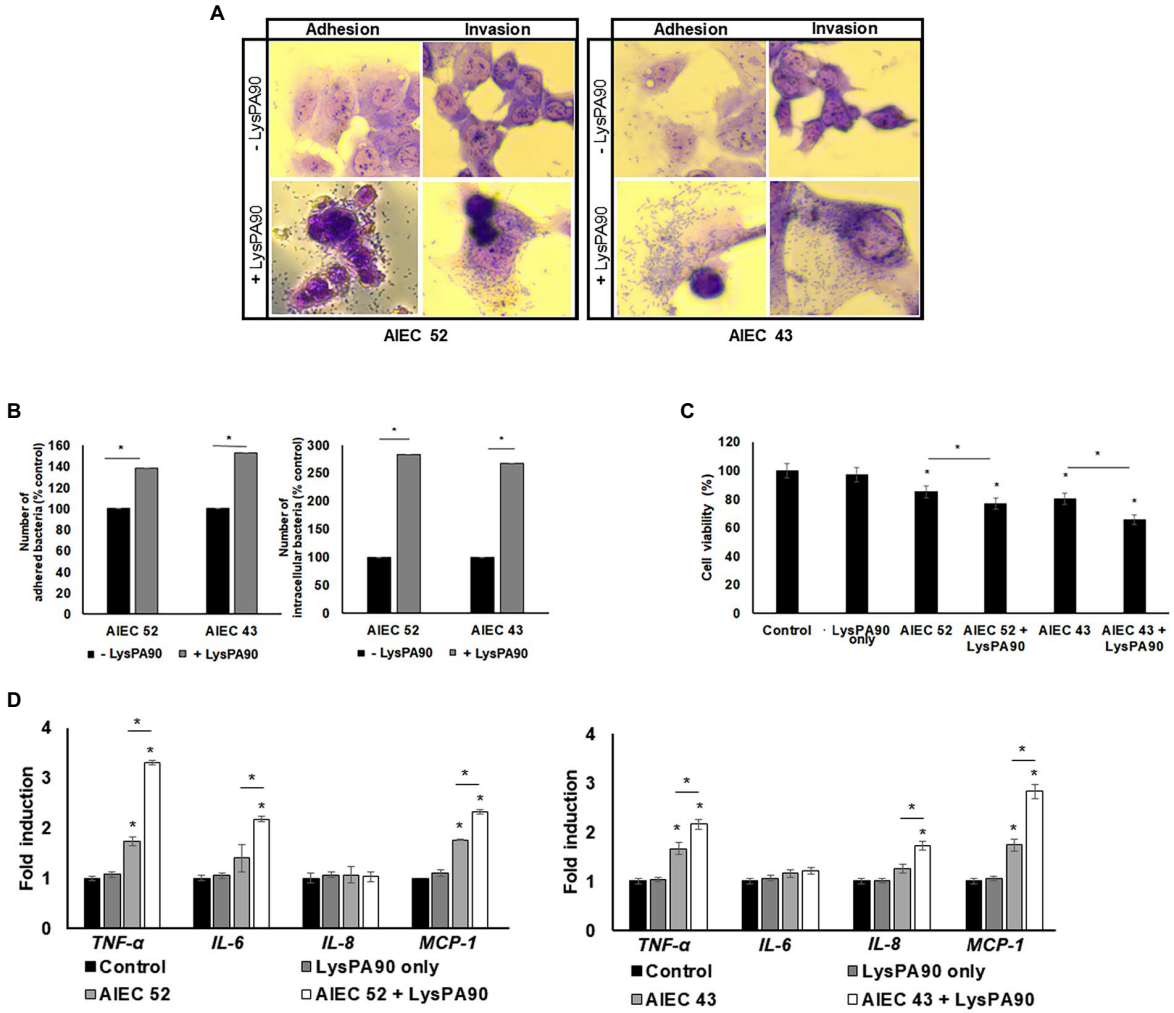
Changes in epithelial Caco-2 cells co-cultured with AIEC strains in the presence of LysPA90. **(A)** Microscopic observation of adhesion or invasion of AIEC strains to Caco-2 cells. Co-cultured AIEC and Caco-2 cells were treated with PBS or LysPA90 to examine any change in the adherence of the bacteria. The culture was further treated with PBS or gentamycin to remove any bacteria outside Caco-2 cells to examine any change in invasion. **(B)** Counting of AIEC cells which adhered to (left) or invaded Caco-2 cells (right). **(C)** MTT assay of Caco-2 cells co-cultured with AIEC strains in the absence or presence of LysPA90. LysPA90 only (without AIEC strains) was used as a control. **(D)** Realtime RT-PCR analysis of gene expression related to inflammation from Caco-2 cells in the presence of AIEC strains and LysPA90. Experiments were performed in triplicate. Data are expressed as mean ± standard deviation.

When epithelial Caco-2 cells were cultured with AIEC strains in the presence or absence of recombinant LysPA90, the number of bacterial cells adhering to the epithelial cells differed visibly under a microscope (Figure 6A). Giemsa staining revealed a crowded population of bacteria on top of, and around Caco-2 cells in the presence of the endolysin. A bacterial count after washing out free bacteria revealed a 40% increase in the AIEC52 strain, and a 50% increase in the AIEC43 strain (Figure 6B). In terms of bacterial invasion, a microscopic analysis after treatment of gentamycin to remove any bacteria outside the cells showed a clear increase in the invading bacterial population in the presence of the endolysin (Figure 6A). Bacterial counts revealed that the presence of the endolysin promoted bacterial invasion 2.8-fold for the AIEC52 strain and 2.7-fold for the AIEC43 strain (Figure 6B). Thus, the influence of the endolysin was greater for invasion than for adhesion. The viability of Caco-2 cells in the presence of AIEC strains decreased and further decreases were seen in the presence of AIEC strains with the addition of LysPA90 (Figure 6C). The expression of the proinflammatory cytokine genes, TNF-α, IL6, and MCP1, from Caco-2 cells was upregulated in the presence of AIEC 43 (Figure 6D), while the expression of the proinflammatory cytokine genes, TNF-α, IL8, and MCP1, from Caco-2 cells was upregulated in the presence of AIEC 52. The expression was further upregulated in the presence of AIEC strains stimulated with LysPA90.

## 4. Discussion

Clinical trials have already been commenced with two recombinant endolysins (identifiers NCT04160468 and NCT03089697 in Clinicaltrials.gov). The majority of drugs are known to impart unexpected adverse effects on hosts, and there is a need to explore any such possible effects of novel endolysin drugs at as early a stage as possible.

The endolysin LysPA90 has intrinsic antibacterial activity when applied as a recombinant protein. This intrinsic antibacterial activity means that endolysin-induced stress for the outer membranes of bacteria seems inevitable, since it passes through the outer membrane to reach the peptidoglycan cell wall. Our data confirmed that the endolysin enters the outer membrane and the resulting damage renders the outer membrane permeable. Since the endolysin penetrates the outer membrane, it may also enter the cytoplasmic membrane by way of the same mechanism seen in antimicrobial peptides, leading to the collapse of membrane potential. Damage to the outer membrane and the cell wall may lead to further stress on the cytoplasmic membrane *via* osmotic pressure which is dependent on the environment encountered by the bacterial cell. Membrane stress has been reported to be related to increases in flagella biosynthesis in *Salmonella enterica* via *rpo*E regulation (Du et al., 2011; Spöring et al., 2018), and this was similarly observed with *E. coli* in this study. In previous studies, many antibiotics have been seen to induce a decrease in motility. However, in this study endolysins were seen to induce membrane stress leading to an increase in flagella biosynthesis, suggesting that bacterial responses to different antibacterials could result in diverse and unanticipated results.

Increased flagella expression has been reported to promote bacterial adhesion and invasion to intestinal epithelial cells by facilitating passage through the surface mucus layer. Flagella expression in AIEC strains, but not in commensal *E. coli*, was reported to be upregulated under intestinal conditions (the presence of bile acid and mucins) (Sevrin et al., 2020). In addition, a deletion mutant of an AIEC strain lacking flagella behaved like a non-pathogenic *E. coli* (Carvalho et al., 2008). In this study, enhanced flagella expression was observed from a commensal *E. coli* strain, induced by a suboptimal concentration of the recombinant endolysin LysPA90. Further enhancements in adhesion and invasion to intestinal epithelial cells were observed from two different AIEC strains induced by the recombinant endolysin. Downregulation of fimbriae expression was also observed in the commensal *E. coli* in RNA-Seq analysis, suggesting increased motility of the bacteria. Nonetheless, the expression of type I fimbriae was not altered by the presence of the endolysin in the AIEC strains. This may suggest that the expression of fimbriae in the AIEC strains was already suppressed, thus facilitating the invasion of AIEC into epithelial cells.

AIECs pass through the mucus layer, enter intestinal epithelial cells, and eventually arrive at the macrophages underneath, leading to inflammation. Active propelling using flagella is one of the methods the bacteria employ to achieve this goal. In this study, *in vitro* cultured Caco-2 cells did not produce mucus, exposing cell surface directly to AIEC. Although the extracellular environment in the absence of mucus layer *in vitro* did not exactly mimic intestinal environment, we still could observe increased adhesion and invasion of AIEC to Caco-2 cells. This is what we could expect from AIECs once passed through mucus layer, encountering intestinal epithelial cells *in vivo*. Endolysin-induced superior motility would permit the surviving bacteria to more actively invade epithelial cells and macrophages, leading to a worsening of inflammation in an intestinal environment. This adverse effect should be considered when using endolysins to treat patients in the future and proper countermeasures explored and utilized.

## Data availability statement

The datasets presented in this study can be found in online repositories. The name of the repository and accession number can be found at: NCBI; MW815133.

## Author contributions

YH, JJ, EK, HH, and MK performed experiments. HY and HM analysed data and wrote manuscript. All authors contributed to the article and approved the submitted version.

## Funding

This work was supported by the Korea National Research Foundation (NRF) Fund (2019M3E5D506666), the Korea Health Industry Development Institute (KHIDI) (HI21C2447), and the HUFS Research Fund of 2022.

## Conflict of interest

YH, HH, MK, and HM are employed by LyseNTech. Co. Ltd.

The remaining authors declare that the research was conducted in the absence of any commercial or financial relationships that could be construed as a potential conflict of interest.

## Publisher’s note

All claims expressed in this article are solely those of the authors and do not necessarily represent those of their affiliated organizations, or those of the publisher, the editors and the reviewers. Any product that may be evaluated in this article, or claim that may be made by its manufacturer, is not guaranteed or endorsed by the publisher.
