# Corrigendum: Motility increase of Adherent Invasive *Escherichia coli* (AIEC) induced by a sub-inhibitory concentration of recombinant endolysin LysPA90

**DOI:** 10.3389/fmicb.2023.1272581

**Published:** 2023-08-15

**Authors:** Yoon Jung Hwang, Jaehak Jo, Eunsuk Kim, Hyunjin Yoon, Hyewon Hong, Min Soo Kim, Heejoon Myung

**Affiliations:** ^1^Department of Bioscience and Biotechnology, Hankuk University of Foreign Studies, Yong-In, Gyung-Gi Do, South Korea; ^2^LyseNTech Co. Ltd., Seongnam, Gyung-Gi Do, South Korea; ^3^Department of Molecular Science and Technology, Ajou University, Suwon, Gyung-Gi Do, South Korea

**Keywords:** bacteriophage, endolysin, flagella, AIEC, membrane stress

In the published article, there was an error in the **Funding** statement. The funding statement for the Korea Health Industry Development Institute (KHIDI) was displayed as: “(HI21C2447010021)”.

The correct Funding statement appears below.

