# Supplementary material for: Motility increase of adherent invasive Escherichia coli (AIEC) induced by a sub-inhibitory concentration of recombinant endolysin LysPA90
Source: Front Microbiol. 2022 Dec 22;13:1093670. doi: 10.3389/fmicb.2022.1093670 (PMC9814724; doi:10.3389/fmicb.2022.1093670)
Supplement: Supplementary file 2 [file Table_2.DOCX]

**Supplementary Table 2**

| **NCBI number** | **Gene name** | **primer** | **sequence (5'-3')** |
| --- | --- | --- | --- |
| **WP_001291603.1** | ***flh*C** | **F** | **gcggtttgttgaaagtggat** |
|  |  | **R** | **gtgggataatatcggcagga** |
| **WP_001259583.1** | ***flh*E** | **F** | **gcctctttctacgcgacaac** |
|  |  | **R** | **ccaggcacttcccagataaa** |
| **WP_000994407.1** | ***fli*F** | **F** | **agctggacttcgccagtaaa** |
|  |  | **R** | **ctgctggcggttattttgat** |
| **WP_000096480.1** | ***flg*K** | **F** | **agcttcacgctgaaaccagt** |
|  |  | **R** | **cccaccgttttactgttgct** |
| **WP_001087467.1** | ***fli*A** | **F** | **gggtgatggaagctatcgaa** |
|  |  | **R** | **accgtttaatggcctgactg** |
| **WP_000079773.1** | ***fli*C** | **F** | **gtatctctctggcgcagacc** |
|  |  | **R** | **agaaccgtttttagccagca** |
| **WP_001295364.1** | ***rpo*E** | **F** | **gtcgtccaccttccagtgat** |
|  |  | **R** | **taaatcttccgggagggact** |
| **WP_000130217.1** | ***rpo*H** | **F** | **ttggcaacctggattcctac** |
|  |  | **R** | **catcaggccgatgttacctt** |
| **WP_000516135.1** | ***dna*K** | **F** | **ggcttcttctggtctgaacg** |
|  |  | **R** | **tagcggctttgtcttcacct** |
| **WP_001118446.1** | ***dna*J** | **F** | **acccagaccggtaagctttt** |
|  |  | **R** | **cgaagctttcttgcagctct** |
| **WP_000520676.1** | ***yde*Q** | **F** | **ccactgacagtagccgacaa** |
|  |  | **R** | **atgactgactggacggttcc** |
| **WP_000830468.1** | ***yeh*D** | **F** | **attattgctgccgctgtctt** |
|  |  | **R** | **tggttggtacagtcggcata** |
| **WP_000033336.1** | ***yfc*V** | **F** | **ggtgcgttcgcttctacttc** |
|  |  | **R** | **cggtgtagaagtcccgttgt** |
| **CCDS4702.1** | ***TNF*-α** | **F** | **cagagggcctgtacctcatc** |
|  |  | **R** | **ggaagacccctcccagatag** |
| **CCDS5375.1** | ***IL-6*** | **F** | **tacccccaggagaagattcc** |
|  |  | **R** | **ttttctgccagtgcctcttt** |
| **CCDS34005.1** | ***IL-8*** | **F** | **gtgcagttttgccaaggagt** |
|  |  | **R** | **ctctgcacccagttttcctt** |
| **CCDS11277.1** | ***MCP-1*** | **F** | **ccccagtcacctgctgttat** |
|  |  | **R** | **tggaatcctgaacccacttc** |
| **CCDS43186.1** | ***OPA-1*** | **F** | **ggccagcaagattagctacg** |
|  |  | **R** | **acaatgtcaggcacaatcca** |
| **CCDS61098.1** | ***DRP1*** | **F** | **acccggagacctctcattct** |
|  |  | **R** | **tgacaacgttgggtgaaaaa** |
| **CCDS9328.1** | ***CDX-2*** | **F** | **agccaagtgaaaaccaggac** |
|  |  | **R** | **tttcctctcctttgctctgc** |
