# Supplementary figures and images for: Motility increase of adherent invasive Escherichia coli (AIEC) induced by a sub-inhibitory concentration of recombinant endolysin LysPA90
Source: Front Microbiol. 2022 Dec 22;13:1093670. doi: 10.3389/fmicb.2022.1093670 (PMC9814724; doi:10.3389/fmicb.2022.1093670)

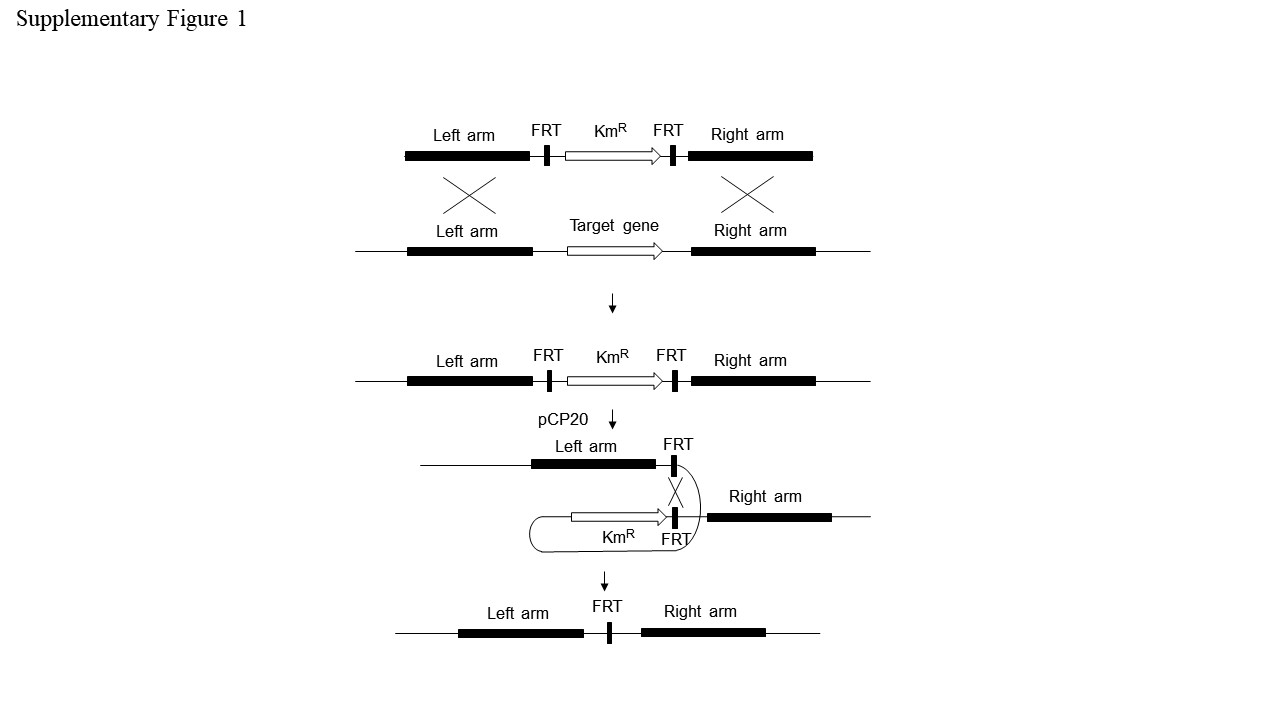

Supplement: Supplementary file 3 [file Image_1.TIF]

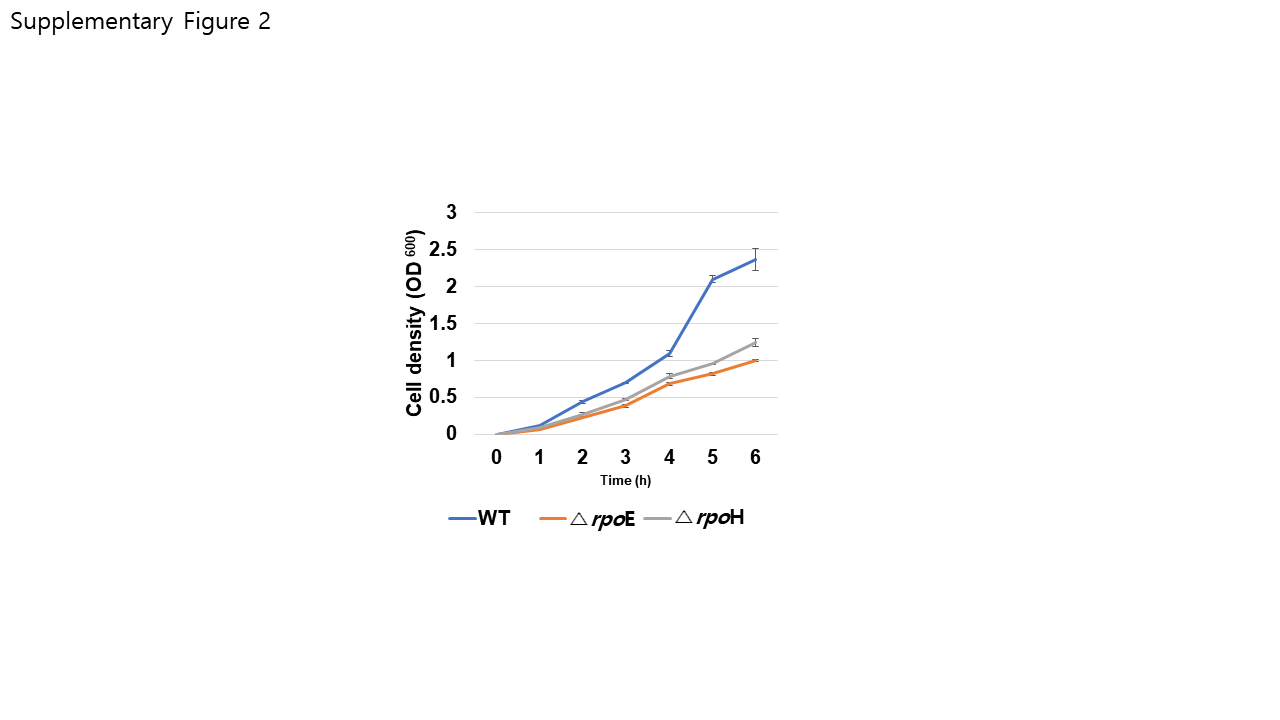

Supplement: Supplementary file 4 [file Image_2.TIF]
